# Challenges and preventive strategies in pediatric drug hypersensitivity reactions: where do we stand?

**DOI:** 10.1186/s13052-025-01888-x

**Published:** 2025-02-11

**Authors:** Angela Klain, Francesca Galletta, Francesca Mori, Irene Bettini, Leonardo Tomei, Antonio Andrea Senatore, Sara Manti, Amelia Licari, Michele Miraglia del Giudice, Cristiana Indolfi

**Affiliations:** 1 Department of Woman, Child and General and Specialized Surgery, University of Campania ’Luigi Vanvitelli’, Naples, 80138 Italy; 2 Pediatric Unit, Department of Human Pathology in Adult and Developmental Age ’Gaetano Barresi’, University of Messina, Messina, 98124 Italy; 3 Allergy Unit, Meyer Children’s Hospital, IRCCS, Florence, 50139 Italy; 4 Pediatric Unit, IRCCS Azienda Ospedaliera-Universitaria di Bologna, Bologna, 40138 Italy; 5 Department of Clinical, Surgical, Diagnostic, and Pediatric Sciences, University of Pavia, Pavia, 27100 Italy; 6 Pediatric Clinic, Fondazione IRCCS Policlinico San Matteo, Pavia, 27100 Italy

**Keywords:** Drug hypersensitivity reactions (DHRs), Prevention, Desensitization, Children

## Abstract

Drug hypersensitivity reactions (DHRs) in pediatric patients present significant clinical challenges due to their potential for severe, life-threatening reactions such as anaphylaxis. Although less common than in adults, DHRs in children are more difficult to diagnose and manage due to limited pediatric-specific research. This study focuses on the role of premedication as a preventive strategy for DHRs, exploring its potential benefits and limitations. While approaches like H1-antihistamines combined with gradual drug dose escalation show promise for reducing mild histamine-mediated reactions, evidence regarding premedication’s effectiveness, particularly for non-IgE-mediated mechanisms, remains inconclusive. Rapid drug desensitization (RDD) protocols demonstrate high success rates in certain cases; however, there are no strong recommendations for or against premedication, and controlled studies documenting its real efficacy are lacking. Further research is crucial to establish clear guidelines and improve the safety and outcomes of pediatric drug allergy management.

**Supplementary Information:**

The online version contains supplementary material available at 10.1186/s13052-025-01888-x.

Dear Editor,

Drug allergies in pediatric patients are a significant concern in clinical practice, as they can lead to severe adverse reactions, including life-threatening anaphylaxis. While drug hypersensitivity reactions (DHRs) are less common in children than in adults, they can be more challenging to diagnose and manage due to limited pediatric-specific data. In light of this, finding preventive strategies for drug allergies in pediatric populations is essential to minimize risks and improve patient quality of life. Premedication (PM) is one of the first preventive approach worth considering, though research on its effectiveness in pediatric DHRs remains limited. Most available studies focus on adult populations and show conflicting results, influenced by factors such as the type of drug, the premedication or desensitization protocol used, and individual patient characteristics. Position papers from the European Network of Drug Allergy (ENDA) and the European Academy for Allergy and Clinical Immunology (EAACI) drug hypersensitivity group indicate that premedication with systemic steroids and antihistamines has limited effectiveness in preventing breakthrough reactions (BRs) [1, 2]. Moreover, premedication may mask early signs of hypersensitivity, creating a false impression of temporary tolerance. However, a recent EAACI position paper suggests that premedication with H1-antihistamines, combined with a slow, gradually increasing dose of certain drugs, may help to mitigate mild reactions due to nonspecific histamine release from mast cells and basophils [3]. This approach could be effective for reactions triggered by agents like opioids, neuromuscular blocking agents (NMBAs), vancomycin, and thiopentone. In this context, taking oral H1-antihistamines 2–3 days before anesthesia may provide effective histamine receptor blockade [3]. Additionally, several factors complicate the use of premedication. Notably, the immunopathological mechanism behind immediate DHRs is not always IgE-mediated. Immediate reactions can also arise through non-IgE pathways, including IgG-mediated mechanisms and complement-mediated responses. For these types of reactions, evidence supporting the efficacy of premedication is even more limited, and few studies have explored its preventive potential. Consequently, understanding and managing both IgE- and non-IgE-mediated reactions remains a challenge in clinical practice. In pediatric patients, the lack of sufficient data has made it impossible to establish recommendations either in favor of or against premedication use.

In this letter, we have reviewed articles on drug adverse reaction prevention in pediatric patients, particularly on the effectiveness of premedication. Most studies focus on premedication in relation to rapid drug desensitization (RDD) procedures, which involve gradually increasing the drug dosage to induce temporary tolerance and prevent hypersensitivity reactions, typically for 2 to 8 h for IgE-mediated reactions; while several days or weeks protocols are necessary for delayed reactions.

In a retrospective study by Enseboga et al. [4], children aged 3 to 13 who experienced HSRs were treated with a modified 12-step RDD protocol, which included premedication with methylprednisolone and hydroxyzine. This approach was used primarily for chemotherapy agents, monoclonal antibodies, and antibiotics, achieving a 90% success rate with no reactions in the majority of desensitization protocols. Positive skin test results were identified as the main risk factor for breakthrough reactions (BRs) [4]. Another study involved 14 pediatric patients who underwent a 12-step desensitization protocol for various drugs, including monoclonal antibodies and antibiotics, with premedication using an antihistamine and methylprednisolone. Mild to severe reactions occurred in only 13% of cases, with all BRs linked to monoclonal antibody use [5]. Dillman et al. [6] explored premedication with corticosteroids and antihistamines in pediatric and adult patients with previous allergic-like reactions to gadolinium-based contrast agents. BRs were primarily observed in patients with a documented history of contrast media reactions, emphasizing the challenges of managing hypersensitivity in such cases [6]. In the case-control studies by van Wassenaer et al. and Szymanska et al. [7, 8], steroid premedication in pediatric IBD patients receiving infliximab was evaluated. Both studies found no significant reduction in infusion reaction rates between groups with and without premedication, suggesting that routine steroid premedication may not be necessary in this population. Yilmaz Topal et al. [9] reviewed records of 211 children receiving biological therapies, finding a 6.64% rate of hypersensitivity reactions, primarily associated with rituximab and infliximab. Some patients required desensitization, which allowed them to safely continue therapy. Premedication with corticosteroids, antihistamines, and additional agents like montelukast was used to mitigate reactions, particularly for those with hematologic-oncologic conditions [9]. These studies collectively highlight the complexity of managing drug allergies in pediatric patients and suggest that while premedication and desensitization protocols can be effective, their success varies depending on the drug type and patient history [10]. However, there are other aspects to consider when discussing drug allergy prevention. Based on the findings in the recent review by the Drug Allergy Commission of the Italian Society of Pediatric Allergy and Immunology (SIAIP), preventive strategies for drug hypersensitivity reactions in children can be developed around key risk factors [11]. Identifying high-risk individuals is essential, with attention to factors such as age, drug history, and any existing comorbidities [11]. Genetic testing is increasingly considered, particularly in children with family histories of DHRs, as certain genetic polymorphisms may increase susceptibility [11]. The approach should prioritize minimizing polypharmacy where possible, as the concurrent use of multiple drugs is a well-known risk factor [11]. Where drug administration is required, the oral route is preferred over intravenous or intramuscular routes to reduce risk [11]. Furthermore, heightened monitoring during periods of viral infections is recommended, as viral co-infections can act as cofactors in triggering drug hypersensitivity [11]. In conclusion, the management of drug allergies in pediatric patients remains complex and requires a comprehensive approach. While premedication and desensitization protocols offer valuable options, their effectiveness varies depending on drug type and individual patient factors. Preventive strategies should be tailored to address high-risk indicators, such as genetic predispositions, polypharmacy, and patient-specific factors like viral infections. Greater interdisciplinary collaboration and more large-scale studies are essential to validate and optimize drug desensitizations in children. Sharing knowledge across pediatric allergy centers will be crucial for advancing this practice, refining these strategies, and improving safety and outcomes in pediatric drug allergy management [12].

## Electronic supplementary material

Below is the link to the electronic supplementary material.

Supplementary Material 1

## Data Availability

Not applicable.

## Abbreviations

DHRs: Drug hypersensitivity reactions
PM: Premedication
ENDA: European Network of Drug Allergy
EAACI: European Academy for Allergy and Clinical Immunology
BRs: Breakthrough reactions
H1: Histamine H1 receptor
NMBAs: Neuromuscular blocking agents
IgE: Immunoglobulin E
IgG: Immunoglobulin G
RDD: Rapid drug desensitization
HSRs: Hypersensitivity reactions
IBD: Inflammatory bowel disease
SIAIP: Italian Society of Pediatric Allergy and Immunology
