# Supplementary material for: Challenges and preventive strategies in pediatric drug hypersensitivity reactions: where do we stand?
Source: Ital J Pediatr. 2025 Feb 11;51:43. doi: 10.1186/s13052-025-01888-x (PMC11817705; doi:10.1186/s13052-025-01888-x)

Napoli, 09 January 2025

**Title:** Challenges and Preventive Strategies in Pediatric Drug Hypersensitivity Reactions: Where Do We Stand?

**Name of the corresponding author:** Angela Klain

Dear Editor,

Please consider the revised letter entitled **"Challenges and Preventive Strategies in Pediatric Drug Hypersensitivity Reactions: Where Do We Stand?",** submitted today to the Italian Journal of Pediatrics.

In this letter, we discuss the prevention and management of drug hypersensitivity reactions (DHRs) in pediatric patients, with a particular focus on the effectiveness of premedication strategies. Our aim is to highlight current evidence, explore challenges associated with both IgE- and non-IgE-mediated reactions, and evaluate the role of premedication and desensitization protocols in reducing hypersensitivity risks. By addressing these aspects, we seek to provide insights into optimizing preventive strategies and improving safety and outcomes in pediatric drug allergy management.

We wish to thank the Editor and the reviewers for their thoughtful comments about the manuscript.

All authors declare that:

- the manuscript has not been published previously and is not under consideration (in whole or in part) for publication elsewhere.
- all Authors approve the submitted manuscript.

Also, all authors declare that they have no relevant conflicts of interest.

I hope this manuscript meets with your approval on behalf of all authors.
Best regards,

Angela Klain

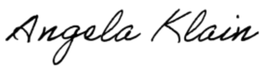

Supplement: Supplementary file 1 — Supplementary Material 1 [file 13052_2025_1888_MOESM1_ESM.pdf]
